# Chemokine receptor co-expression reveals aberrantly distributed T_H_ effector memory cells in GPA patients

**DOI:** 10.1186/s13075-017-1343-8

**Published:** 2017-06-14

**Authors:** Lucas L. Lintermans, Abraham Rutgers, Coen A. Stegeman, Peter Heeringa, Wayel H. Abdulahad

**Affiliations:** 1 Department of Rheumatology and Clinical Immunology, University of Groningen, University Medical Center Groningen, Hanzeplein 1, 9713 GZ Groningen, The Netherlands; 2 Department of Internal Medicine, Division of Nephrology, University of Groningen, University Medical Center Groningen, Hanzeplein 1, 9713 GZ Groningen, The Netherlands; 3 Department of Pathology and Medical Biology, University of Groningen, University Medical Center Groningen, Hanzeplein 1, 9713 GZ Groningen, The Netherlands

**Keywords:** Vasculitis, Granulomatosis with polyangiitis, T_H_ cells, Effector memory T_H_ cells, Chemokine receptors

## Abstract

**Background:**

Persistent expansion of circulating CD4^+^ effector memory T cells (T_EM_) in patients with granulomatosis with polyangiitis (GPA) suggests their fundamental role in disease pathogenesis. Recent studies have shown that distinct functional CD4^+^ T_EM_ cell subsets can be identified based on expression patterns of chemokine receptors. The current study aimed to determine different CD4^+^ T_EM_ cell subsets based on chemokine receptor expression in peripheral blood of GPA patients. Identification of particular circulating CD4^+^ T_EM_ cells subsets may reveal distinct contributions of specific CD4^+^ T_EM_ subsets to the disease pathogenesis in GPA.

**Method:**

Peripheral blood of 63 GPA patients in remission and 42 age- and sex-matched healthy controls was stained immediately after blood withdrawal with fluorochrome-conjugated antibodies for cell surface markers (CD3, CD4, CD45RO) and chemokine receptors (CCR4, CCR6, CCR7, CRTh2, CXCR3) followed by flow cytometry analysis. CD4^+^ T_EM_ memory cells (CD3^+^CD4^+^CD45RO^+^CCR7^-^) were gated, and the expression patterns of chemokine receptors CXCR3^+^CCR4^-^CCR6^-^CRTh2^-^, CXCR3^-^CCR4^+^CCR6^-^CRTh2^+^, CXCR3^-^CCR4^+^CCR6^+^CRTh2^-^, and CXCR3^+^CCR4^-^CCR6^+^CRTh2^-^ were used to distinguish T_EM_1, T_EM_2, T_EM_17, and T_EM_17.1 cells, respectively.

**Results:**

The percentage of CD4^+^ T_EM_ cells was significantly increased in GPA patients in remission compared to HCs. Chemokine receptor co-expression analysis within the CD4^+^ T_EM_ cell population demonstrated a significant increase in the proportion of T_EM_17 cells with a concomitant significant decrease in the T_EM_1 cells in GPA patients compared to HC. The percentage of T_EM_17 cells correlated negatively with T_EM_1 cells in GPA patients. Moreover, the circulating proportion of T_EM_17 cells showed a positive correlation with the number of organs involved and an association with the tendency to relapse in GPA patients. Interestingly, the aberrant distribution of T_EM_1 and T_EM_17 cells is modulated in CMV- seropositive GPA patients.

**Conclusions:**

Our data demonstrates the identification of different CD4^+^ T_EM_ cell subsets in peripheral blood of GPA patients based on chemokine receptor co-expression analysis. The aberrant balance between T_EM_1 and T_EM_17 cells in remission GPA patients, showed to be associated with disease pathogenesis in relation to organ involvement, and tendency to relapse.

**Electronic supplementary material:**

The online version of this article (doi:10.1186/s13075-017-1343-8) contains supplementary material, which is available to authorized users.

## Background

Granulomatosis with polyangiitis (GPA) is a severe systemic autoimmune disease of unknown etiology. The hallmark of the disease is the presence of antineutrophil cytoplasmic autoantibodies (ANCAs) mainly directed against protienase-3 (PR3) [1]. GPA is characterized by necrotizing granulomatosis in the respiratory tract, and a systemic vasculitis preferentially affecting pulmonary and renal small- and medium-sized blood vessels. The abundance of T cells in these vasculitic and granulomatous lesions of GPA patients support their involvement in disease pathogenesis [2]. There is substantial evidence of activated T cells and antigen-driven T cell responses in GPA [3–5] In addition, remission has been induced with therapeutics directed against T cells in patients with refractory GPA [6, 7]. These studies strongly indicate a T cell-mediated pathology in this disease.

The involvement of cluster of differentiation (CD)4^+^ T helper (T_H_) cells in the pathogenesis of GPA has been suggested to depend on disease activity, and whether the disease is localized, i.e. restricted to the respiratory tract, or generalized. Prior to the discovery of T_H_17 cells, research in GPA focused on the disturbed balance between T_H_1 and T_H_2 cells. It was found that GPA patients with active disease demonstrated a dysregulated cytokine prolife of circulating T cells with increased IFN-γ production versus a normal interleukin (IL)-4 production [8]. Additional studies demonstrated the presence of T_H_1-associated markers in the circulation as well as in nasal granulomatous lesions of patients with localized disease, while T_H_2-associated markers were dominant in generalized disease [9–11]. More recently, levels of IL-17A, the T_H_17-associated cytokine, were found to be elevated in serum of GPA patients irrespective of active or quiescent disease [12]. In addition, a relative increase in autoantigen-specific T_H_17 cells in GPA patients has been reported [12, 13].

Defects in regulatory T cell (T_REG_) function in GPA patients may contribute to abnormal skewing in T_H_ cell responses and may result in an expansion of the CD4^+^ effector memory T (CD4^+^ T_EM_) cell population [14]. In addition, altered T_H_ cell distribution in GPA patients may be in part driven by chronic cytomegalovirus (CMV) infection [15]. We have demonstrated previously that circulating CD4^+^ T_EM_ cells (CCR7^-^CD45RO^+^) in GPA patients were proportionally increased during remission [16], but were decreased during renal active disease upon migration to the inflammatory site [17]. However, during active renal disease not all circulating CD4^+^ T_EM_ cells tend to migrate to the target tissues [17]. It is possible that a subset of circulating CD4^+^ T_EM_ cells have a distinct migratory capacity and pathogenic function in GPA patients related to distinct clinical manifestations.

The recruitment of the CD4^+^ T_EM_ cells to inflammatory sites is orchestrated by their chemokine receptors. Analysis of chemokine receptor expression has been instrumental in the characterization of memory T_H_ subsets with distinct cytokine patterns and antigen responses [18]. The expression pattern of four chemokine receptors allows the identification of CD4^+^ T_EM_ subsets, which are defined as T_EM_1 [C-C chemokine receptor (CCR)6^-^ CXC chemokine receptor 3 (CXCR3)^+^CCR4^+^CRTh2^-^] T_EM_2 (CCR6^-^CXCR3^-^CCR4^+^CRTh2^+^), T_EM_17 (CCR6^+^CXCR3^-^CCR4^+^CRTh2^-^) [19, 20], and a subset that exhibits both T_H_17 and T_H_1 features, referred to as T_EM_17.1 (CCR6^+^CXCR3^+^CCR4^-^ CRTh2^-^) [21, 22].

The aim of the present study was to determine the distribution of circulating CD4^+^ T_EM_ cell subsets based on chemokine receptor expression in GPA patients. Identification of particular circulating CD4^+^ T_EM_ subsets may reveal distinct associations of specific CD4^+^ T_EM_ subsets with clinical manifestations or with autoantibodies in GPA patients.

## Methods

### Study population

Peripheral blood was collected from 63 GPA patients in remission (r-GPA) and 42 age- and sex-matched healthy controls (HCs) in a cross-sectional study..The r-GPA patients fulfilled the criteria of the American College of Rheumatology and the Chapel Hill Consensus Conference definition for GPA [23, 24]. Only patients with PR3-ANCA positivity at diagnosis, and complete remission of their disease at the time of sampling, were included in the study. The PR3-ANCA titers were measured by indirect immunofluorescence (IIF) on ethanol-fixed human granulocytes according to the standard procedure as described previously [25]. ANCA titers lower than 1:20 were considered negative. Complete remission was defined as the complete absence of clinical signs and symptoms of active vasculitis, as indicated by a score of zero on the Birmingham Vasculitis Activity Score (BVAS) [26]. According to these criteria, blood samples were taken during a visit to our outpatient clinic.

Disease extent was defined as localized when GPA was restricted to the upper and lower respiratory tract and generalized when systemic disease with vasculitis extended to more clinical manifestations including involvement of kidneys, joints, eye, and nervous system. None of the patients and controls experienced an infection at the time of sampling.

Twenty-nine of 63 r-GPA patients were treated with maintenance immunosuppressive therapy at time of blood sampling. Three r-GPA patients received azathioprine, 12 r-GPA patients received azathioprine in combination with prednisolone, six r-GPA patients were treated with low-dose prednisolone, seven r-GPA patients received low-dose prednisolone in combination with mycophenolate mofetil (MMF), and one r-GPA patient was treated with methotrexate.

Detailed clinical and laboratory characteristics of the patients are summarized in Table 1. All patients and healthy volunteers provided informed consent and the local medical ethics committee approved the study.

**Table 1:** Laboratory and clinical characteristics of r-GPA patients and HC

	r-GPA	HC
Subjects, *n* (% male)	63 (% 44)	42 (% 40)
Age, mean (range)	62.3 (26.8–85.2)	57.2 (21.5–86.8)
PR3-ANCA^a^, *n* (% positive)	39 (% 62)	
PR3-ANCA titer, median (range)	1:40 (0–1:640)	
Creatinine umol/L, median (range)	86 (52–224)	
CRP mg/L, median (range)	2.7 (0.3–99)	
eGFR ml/min*1.73 m^2^, median (range)	64 (21–109)	
CMV seropositive, *n* (% positive) (N.D.)	33 (% 54) (2)	21 (% 58) (6)
*S. aureus*, *n* (% positive) (N.D.)	27 (% 44) (1)	
BVAS, mean	0	
Disease duration in years, median (range)	9.6 (1.9–42.7)	
No. of total relapses, median (range)	1 (0–7)	
Relapser^b^, *n* (%)	43 (% 68)	
Disease type, *n* (% generalized)	52 (% 83)	
Treatment at time of sampling, *n* (%)		
Azathioprine	3 (% 5)	
Azathioprine + prednisolone	12 (% 19)	
Prednisolone	6 (% 10)	
Mycophenolate mofetil + prednisolone	7 (% 11)	
Methotrexate	1 (% 2)	
No immunosupressive treatment	34 (% 54)	
Co-trimoxazole, high dose/low dose/no dose	17/15/31	
No. of organs involved, median (range)	3 (1–7)	
Clinical manifestations, *n* (%)		
Renal	35 (% 56)	
ENT	45 (% 71)	
Joints	36 (% 57)	
Pulmonary	40 (% 63)	
Nervous system	20 (% 32)	
Eyes	24 (% 38)	
Cutaneous	13 (% 21)	
Other	7 (% 11)	

Characteristics at sampling time point

*BVAS* Birmingham Vasculitis Activity Score, *CMV* cytomegalovirus, *CRP* C-reactive protein, *eGFR* estimated glomerular filtration rate, *ENT* ear, nose and throat, *GPA* granulomatosis with polyangiitis, *HC* healthy control, *PR3-ANCA* antineutrophil cytoplasmic antibodies targeting proteinase 3, *r-GPA* GPA patient in remission, *S. aureus Staphylococcus aureus*

^a^ANCA-positive titer ≥1:40, ANCA-negative ≤1:20

^b^Relapser: GPA patient that had ≥1 relapse after diagnosis until time of sampling

### Sample preparation and immunophenotyping by flow cytometry

EDTA-anticoagulated peripheral blood was obtained by venipuncture from r-GPA patients and HCs. Whole blood samples were stained within 4 hours after blood withdrawal with appropriate concentrations of fluorochrome-conjugated monoclonal antibodies for cell surface antigens. The samples were immediately processed to obtain the most sensitive detection for the chemokine receptor expression and to minimize cell manipulation. The peripheral blood was stained using the following monoclonal antibodies for cell surface antigens in combination: Alexa Fluor® 700-conjugated anti-CD3, eFluor® 450 (eF450)-conjugated anti-CD4 (both from eBioscience, San Diego, CA, USA), fluorescein isothiocyanate (FITC)-conjugated anti-CD45RO, phycoerythrin-cyanin7 (PE-C7)-conjugated anti-CCR7 (both from BD Biosciences, Franklin Lakes, NJ, USA), PE-conjugated anti-CRTh2, allophycocyanin-C7 (APC-Cy7)-conjugated anti-CXCR3, peridin chlorophyll α-protein (PerCP-Cy5.5-conjugated anti-CCR4, and Brilliant Violet 605™ (BV605)-conjugated anti-CCR6 (all from BioLegend, San Diego, CA, USA). The appropriated isotype-matched control antibodies of irrelevant specificity were added to a separate tube as negative controls. Samples were incubated for 15 minutes at room temperature. Afterward, cells were treated with 2 mL diluted FACS lysing solution (BD Biosciences) for 10 minutes. Finally, the samples were washed in PBS containing 1% (w/v) bovine serum albumin (BSA), and immediately analyzed by eight-color flow cytometric analyses on BD™ LSR II flow cytometer. Data were collected for 1.0 *10^6^ events for each sample and plotted using Kaluza v1.2 (Beckman Coulter, Brea, CA, USA). Lymphocytes were gated for analysis based on forward and side scatter properties. Positively and negatively stained populations were calculated by quadrant dot-plot analysis or histograms, determined by the appropriate isotype controls. Within the CD4^+^ T_EM_ cell subset (CD4^+^CCR7^-^CD45RO^+^) the expression pattern of chemokine receptors CCR6^-^CXCR3^+^CCR4^-^CRTh2^-^, CCR6^-^CXCR3^-^CCR4^+^CRTh2^+^, CCR6^+^CXCR3^-^CCR4^+^CRTh2^-^, and CCR6^+^CXCR3^+^CCR4^-^CRTh2^-^ were used to distinguish T_EM_1, T_EM_2, T_EM_17, and T_EM_17.1 cells, respectively.

### Detection of *S. aureus*

From 62 r-GPA patients, *S. aureus* nasal carriers were determined as described previously [27]. Briefly, *S. aureus* nasal isolates were sampled by rotating a sterile cotton swab in each anterior nary. Swabs were inoculated on 5% sheep-blood and salt mannitol agar for 72 h at 35 °C. *S. aureus* was identified by coagulase and DNase positivity. Patients were considered to be chronic nasal carriers when ≥50% of their nasal cultures grew *S. aureus.*

### CMV ELISA

CMV-specific IgG was determined in serum samples using an in-house enzyme-linked immunosorbent assay (ELISA). In brief, 96-well ELISA plates (Greiner, Kremsmünster, Austria) were coated overnight with lysates of CMV-infected fibroblasts. Lysates of non-infected fibroblasts were used as negative controls. Following coating, serial (1:100–1:3200) dilutions of serum samples were incubated for 45 minutes. Next, goat anti-human IgG-HRP (Southern Biotech, Birmingham, AL, USA) was added and incubated for 45 minutes. Samples were incubated with TBE substrate (Sigma-Aldrich, St. Louis, MO, USA) for 15 minutes and the reaction was stopped with sulfuric acid. The plates were scanned on a Versamax reader (Molecular Devices, Sunnyvale, CA, USA). A pool of sera from three CMV-seropositive individuals with known concentrations of CMV-specific IgG was used to quantify levels of CMV-specific IgG in the tested samples.

### Statistical analysis

Statistical analysis was performed using SPSS v22 (IBM Corporation, Armonk, NY, USA) and GraphPad prism v5.0 (GraphPad Software, San Diego, CA, USA). Data are presented as median values. Data were analyzed with the D’Agostino-Pearson omnibus normality test for Gaussian distribution. For comparison between r-GPA patients and HCs the unpaired *t* test was used for data with Gaussian distribution and the Mann-Whitney *U* test for data without Gaussian distribution. For intra-individual comparison of values at multiple time points during follow-up, repeated measures analysis of variance was used if data were normally distributed and a Friedman test was used if data had a non-Gaussian distribution. The association between clinical parameters and CD4^+^ T_EM_ cell subsets in inclusion samples of r-GPA patients was investigated using the Spearman’s rank correlation coefficient. In order to account for interactions of CMV and age on the percentage of CD4^+^T cells subsets and CD4^+^T_EM_ cell subsets we used a linear (Enter) regression analysis. Non-normally distributed data were log-transformed. Differences were considered statistically significant at two-sided *p* values equal to or less than 0.05.

## Results

### Higher frequency of CD4^+^ T_EM_ cells in peripheral blood of GPA patients in remission

**Fig. 1:**
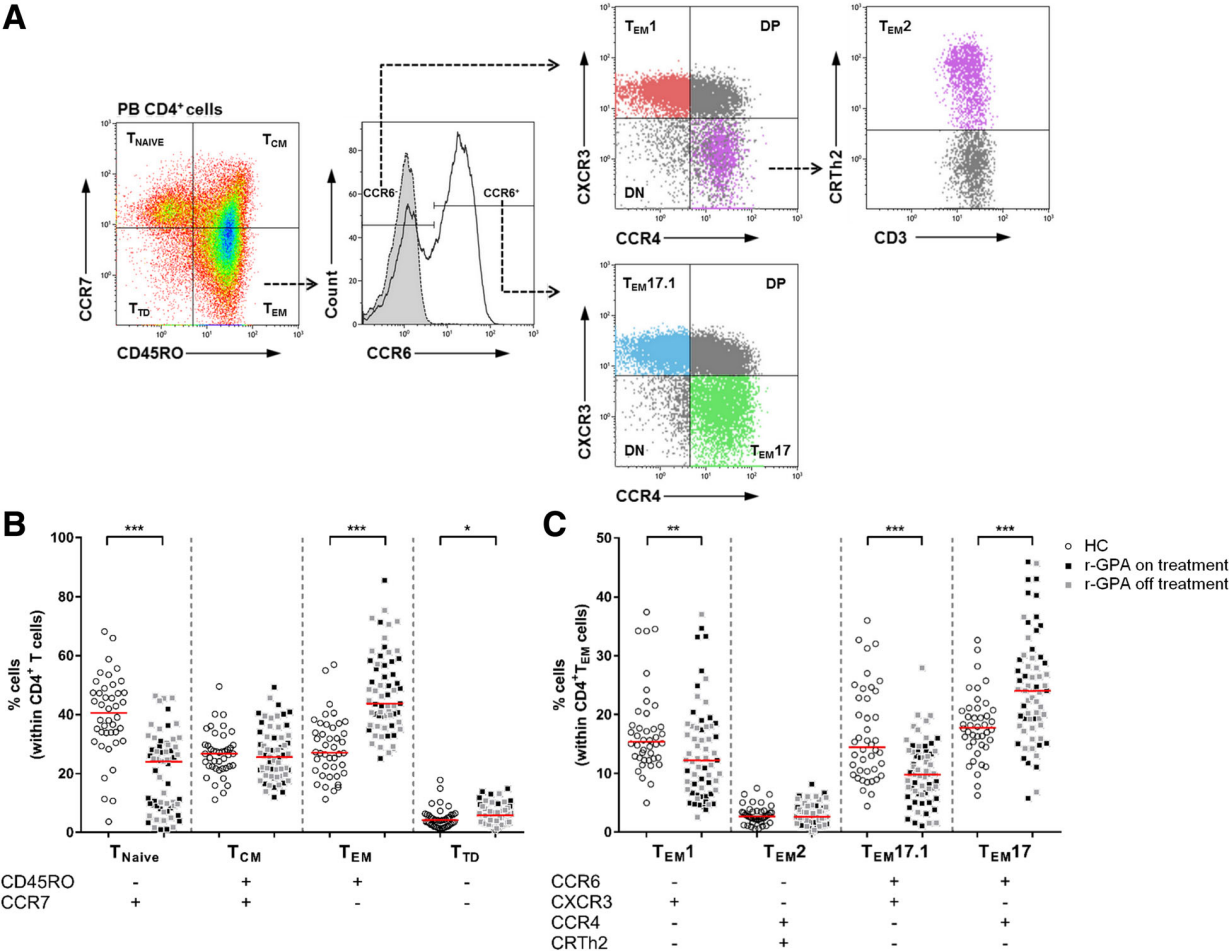
T cell subset distribution between HC and r-GPA patients. **a** Gating strategy of CD4^+^ T_EM_ cell subsets using chemokine receptor expression patterns. The flow cytometry plots show sequential gating (*dashed arrows*) to identify different CD4^+^ T_EM_ cell subsets within the CD4^+^T_EM_ cell population. CD4^+^ T cell subsets from peripheral blood were identified based on the expression of CCR7 and CD45RO. Within CD4^+^T_EM_ cells CCR6^-^ and CCR6^+^ cells were identified based on the isotype (*grey histogram with dashed line*). Within CCR6^-^ CD4^+^T_EM_ cells expression of CXCR3 and CCR4 was analyzed to identify T_EM_1 cells (CD4^+^CD45RO^+^CCR7^-^CCR6^-^CXCR3^+^CCR4^-^). Expression of CRTh2 was used to identify lineage-committed T_EM_2 cells (CD4^+^CD45RO^+^CCR7^-^CCR6^-^CXCR3^-^CCR4^+^CRTh2^+^) derived from CCR6^-^CXCR3^-^CCR4^+^ CD4^+^T_EM_ cells. The CXCR3 and CCR4 expression was also analyzed on CCR6^+^ CD4^+^T_EM_ cells to identify T_EM_17 cells (CD4^+^CD45RO^+^CCR7^-^CCR6^+^CXCR3^-^CCR4^+^), and T_EM_17.1 cells (CD4^+^CD45RO^+^CCR7^-^CCR6^+^CXCR3^+^CCR4^-^). The unclassified populations within the CCR6^-^ and CCR6^+^ populations were identified, that were double-negative (*DN*) or double-positive (*DP*) for CXCR3 and CCR4. Representative flow cytometry plots from one r-GPA patient. **b** Percentages of CD45RO^-^CCR7^+^ (*T*_*NAIVE*_), CD45RO^+^CCR7^+^ (*T*_*CM*_), CD45RO^+^CCR7^-^ (*T*_*EM*_) and CD45RO^-^CCR7^-^ (*T*_*TD*_) subpopulations within the CD4^+^ T cell population in peripheral blood of HCs (*open circles*; *n* = 42) and r-GPA patients (*filled squares*; *n* = 63). **c** The percentage of CD4^+^ T_EM_ cell subsets in peripheral blood of HCs (*open circles*; *n* = 42) and r-GPA patients (*filled squares*; *n* = 63). *Black squares* indicate r-GPA patients on treatment (*n* = 29), *grey squares* indicate r-GPA patients off treatment (*n* = 34). *Horizontal lines* represent median percentages. ^*^ *p* < 0.05, ^**^ *p* < 0.01, and ^***^ *p* < 0.001. *CCR* C-C chemokine receptor, *CD* cluster of differentiation, *CXCR3* CXC chemokine receptor 3, *HC* healthy control, *r-GPA* GPA patient in remission, *T*_*EM*_ effector memory T cell

We have previously reported that r-GPA patients have an increased percentage of circulating CD4^+^ T_EM_ cells compared to HC [16]. Here, we confirm that within the CD4^+^ T cell population in the peripheral blood of r-GPA patients the frequency of CD4^+^ T_EM_ cells was significantly higher compared to HCs (Fig. 1b). In addition, the frequency of CD4^+^ T_Naïve_ cells was significantly lower in r-GPA patients compared to HCs, whereas the proportions of CD4^+^ T_CM_ cells did not differ between r-GPA patients and HCs. The proportions of CD4^+^ T_TD_ cells were higher in r-GPA compared to HCs.

### Increased frequency of CD4^+^T_EM_17 and decreased frequency of CD4^+^T_EM_1 in peripheral blood of patients with GPA

Having demonstrated a significant increase in the frequency of CD4^+^ T_EM_ cells in r-GPA patients we next zoomed in on the phenotypic distribution within this expanded population. As mentioned earlier, CD4^+^ T_EM_ cell population can be subdivided into four T_EM_ subsets based on their differential expression of the chemokine receptors CCR6, CCR4, CXCR3 and CRTh2. We applied a chemokine receptor gating strategy, as shown in Fig. 1a, to identify the distribution of circulating T_EM_1 (CCR6^-^CXCR3^+^CCR4^-^CRTh2^-^), T_EM_2 (CCR6^-^CXCR3^-^CCR4^+^CRTh2^+^), T_EM_17 (CCR6^+^CXCR3^-^CCR4^+^CRTh2^-^), and T_EM_17.1 (CCR6^+^CXCR3^+^CCR4^-^CRTh2^-^) cell subsets among the CD4^+^ T_EM_ cells. The analysis demonstrated a significant decrease in the frequency of T_EM_1 and T_EM_17.1 cells in r-GPA patients compared to HCs (Fig. 1c). The frequencies of T_EM_17 cells were significant higher in r-GPA compared to HCs. No statistical significant difference was reached for the distribution of T_EM_2 cells between r-GPA patients and HCs. In addition, no changes were observed in the percentages of both T_EM_1, and T_EM_17 subsets in three consecutive samples during 6 months of follow-up in individual r-GPA patients (data not shown). Furthermore, we observed CXCR3^+^CCR4^+^ (double positive, DP) and CXCR3^-^CCR4^-^ (double negative, DN) CCR6^+^ T_EM_ cells. Although little is known about the function of these cells, it has been described that the DP CCR6^+^ T_EM_ cells produce low levels of IL-17A and RORC with intermediate IFN-γ and T-bet levels [28]. In our study, we did not observe differences in these unclassified DP and DN population of either CCR6^+^ or CCR6^-^ CD4^+^T_EM_ cells between r-GPA patients and HCs (data not shown).

### The frequency of T_EM_17 cells negatively correlates with the frequency of T_EM_1 cells in peripheral blood of r-GPA patients

**Fig. 2:**
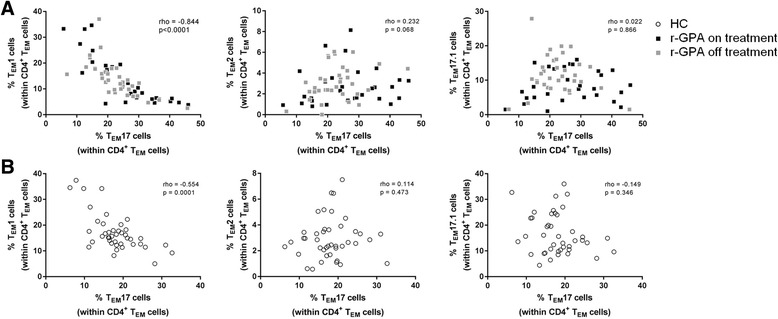
T_EM_17 cells negatively correlate with T_EM_1 cells. Correlation between the percentage of T_EM_17 cells and T_EM_1 (*left*), T_EM_2 (*middle*) and T_EM_17.1 (*right*) cells within the CD4^+^T_EM_ cell population of **a** r-GPA patients (*filled squares*; *n* = 63), *black squares* patients on treatment (*n* = 29) and *grey squares* patients off treatment (*n* = 34) and **b** HC (*open circles*; *n* = 42). Correlations were determined by Spearman’s correlation coefficient (rho) and the level of significance is indicated by the *p* value. *CD* cluster of differentiation, *HC* healthy control, *r-GPA* GPA patient in remission, *T*_*EM*_ effector memory T cell

In vitro and in vivo studies have provided evidence that T_H_1 and T_H_17 cell responses counterregulate each other during disease development in GPA [13, 29]. Therefore, we investigated whether the increased proportion of T_EM_17 cells correlated with the decreased proportion of T_EM_1 cells. As shown in Fig. 2, a significant negative correlation between decreased proportions of T_EM_1 cells and increased proportions of T_EM_17 cells was observed in r-GPA patients (Spearman’s rho = -0.844, *p* < 0.0001). However, neither T_EM_2 cells nor T_EM_17.1 cells correlated significantly with T_EM_17 cells (Fig. 2a). Similar observations were found for HCs, although the correlation between T_EM_1 and T_EM_17 cells (Spearman’s rho = −0.554) was less pronounced in comparison to r-GPA patients (Spearman’s rho = −0.844) (Fig. 2b).

### Immunosuppressive therapy and the imbalance in CD4^+^ T_EM_ cell subsets

To address the question whether current immunosuppressive treatment influences the imbalance in T_EM_1 and T_EM_17 cell subsets, r-GPA patients were separated into patients not receiving immunosuppressive treatment (off treatment; n = 34), and patients that did receive immunosuppressive treatment (on treatment; *n* = 29) (Fig. 1c). No significant differences were observed in the frequencies of T_EM_1 cells between the untreated and treated r-GPA patients (median 12.3%, interquartile rang 9.5–17.8% vs 9.0%, 5.6–19.2%). In addition, no significant differences were detected in the frequencies of T_EM_17 cells between untreated and treated r-GPA patients (22.3%, 18.0–26.5% vs 26.5%, 17.2–35.4%). Therefore, immunosuppressive treatment at time of sampling was not responsible for the imbalances observed between the T_EM_1 and T_EM_17 cell subsets.

### The influence of *S. aureus* and CMV infection on the frequencies of circulating T_EM_1 and T_EM_17 cells in GPA patients

**Fig. 3:**
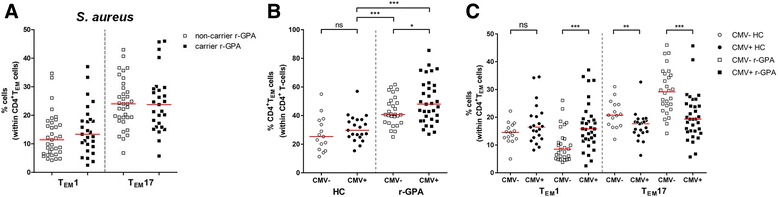
T_EM_1 and T_EM_17 cell distribution between r-GPA patients with *S. aureus* nasal carriage and latent CMV infection. **a** The circulating percentage of T_EM_1 and T_EM_17 cells within the CD4^+^T_EM_ cell population in peripheral blood of *S. aureus*-non-carrying r-GPA patients (*open squares*; *n* = 35) and carrying r-GPA patients (*filled squares*; *n* = 27). **b** The circulating percentage of CD4^+^T_EM_ cells within the CD4^+^ T cell population in peripheral blood, and **c** the circulating percentage of T_EM_1 and T_EM_17 cells within the CD4^+^T_EM_ cell population in peripheral blood of HC CMV-seronegative (*open circles*; *n* = 15), CMV-seropositive (*filled circles*; *n* = 21), r-GPA patients CMV-seronegative (*open squares*; *n* = 28), and CMV-seropositive (*filled squares*; *n* = 33). *Horizontal lines* represent median percentages. ^*^ *p* < 0.05, ^**^ *p* < 0.01, and ^***^ *p* < 0.001. *CD* cluster of differentiation, *CMV* cytomegalovirus, *HC* healthy control, *ns* not significant, *r-GPA* GPA patient in remission, *S. aureus*, *Staphylococcus aureus*, *T*_*EM*_ effector memory T cell

Physiologically, T_H_17 cells are important in the defense against bacterial infection (e.g. *Staphylococcus aureus* (*S. aureus*)), by IL-17-mediated activation of neutrophils. Interestingly, chronic nasal carriage of *S. aureus* has been suggested to drive the T_H_17 response in ANCA-associated vasculitis (AAV) [30]. Therefore we investigated whether chronic nasal carriage of *S. aureus* influenced the proportions of T_EM_1 and T_EM_17 cells. No significant differences were found in the proportion of T_EM_1 and T_EM_17 cells between r-GPA patients with or without *S. aureus* nasal carriage (Fig. 3a), even after excluding co-trimoxazole treatment (data not shown).

Besides bacterial infections, latent viral infection can also influence the T cell compartment in humans. The expansion in CD4^+^ T_EM_ cells in GPA patients has been suggested to be driven by latent cytomegalovirus (CMV) [15]. Here, we found a slight increase in the percentage of circulating CD4^+^T_EM_ cells of CMV-seropositive compared to CMV-seronegative populations. This difference was statistically significant in r-GPA patients (*p* = 0.044), but not in HCs (*p* = 0.234) (Fig. 3b). In addition, both CMV-seropositive and CMV-seronegative r-GPA patients showed significantly increased percentages of CD4^+^T_EM_ cells compared to CMV-seropositive HCs.

To investigate the possibility that the shift from T_H_1 toward T_H_17 cells in GPA patients was the result of CMV carriage, we compared the proportions of T_EM_1 and T_EM_17 cells between CMV-seropositive and CMV-seronegative r-GPA patients. As shown in Fig. 3c, CMV-seropositive r-GPA patients demonstrated significantly higher frequencies of T_EM_1 cells and significantly lower frequencies of T_EM_17 cells compared to CMV-seronegative r-GPA patients. In contrast, CMV serostatus in HCs did not change the proportions of T_EM_1 cells but a small decrease in the percentage of T_EM_17 cells in CMV-seropositive HCs compared to CMV-seronegative HCs was observed.

Furthermore, CMV-specific serum IgG levels in r-GPA patients showed a positive correlation with the percentage of T_EM_1 cells (Spearman’s rho = 0.408, *p* < 0.001) and a negative correlation with the percentage of T_EM_17 cells (Spearman’s rho = −0.468, *p* < 0.0001).

### Association of T_EM_1 and T_EM_17 frequencies with laboratory and clinical parameters

We next explored whether disturbed frequencies in T_EM_1 and T_EM_17 cells correlated with various laboratory and clinical parameters of r-GPA patients (Table 2). Serum ANCA titers in GPA patients have often been related to disease activity and risk of relapse. Therefore, we investigated the relation between ANCA titer at time of sampling and the proportions of T_EM_1 and T_EM_17 cells in r-GPA patients. No correlation was observed between ANCA titers and the frequencies of either T_EM_1 cells or T_EM_17 cells. In addition, no correlations were found between frequencies of either T_EM_1 cells or T_EM_17 cells and any other laboratory parameter measured at the time of inclusion, including creatinine levels, C-reactive protein (CRP) serum levels, and epidermal growth factor receptor (eGFR).

**Table 2:** Associations of T_EM_1 cell and T_EM_17 cell percentages with clinical parameters

Clinical parameters	% T_EM_1 cells		% T_EM_17 cells	
	Spearman’s rho	*p* value	Spearman’s rho	*p* value
PR3-ANCA titer	0.026	0.839	−0.048	0.707
Creatinine (umol/L)	−0.039	0.759	0.164	0.198
CRP (mg/L)	0.047	0.715	0.015	0.909
eGFR (mL/min*1.73 m^2^)	−0.079	0.540	−0.006	0.962
Disease duration (years)	−0.023	0.857	0.063	0.626
No. of total relapses	−0.148	0.246	0.181	0.155
No. of organs involved	−0.264^*^	0.037	0.390^**^	0.002

*CRP* C-reactive protein, *eGFR* estimated glomerular filtration rate, *PR3-ANCA* antineutrophil cytoplasmic antibodies targeting proteinase 3, *TEM* effector memory T cell

* *p* < 0.05 and ** *p* < 0.01

**Fig. 4:**
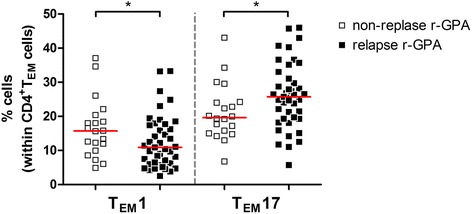
T_EM_1 and T_EM_17 cell distribution between relapsing and non-relapsing r-GPA patients. The circulating frequencies of T_EM_1 and T_EM_17 cells within the CD4^+^T_EM_ cell population in peripheral blood of non-relapsing r-GPA patients (*open squares*; *n* = 20) and r-GPA patients that experienced ≥ 1 relapse during their disease course until inclusion in this study (*filled squares*; *n* = 43). *Horizontal lines* represent median percentages. ^*^ *p* < 0.05. *CD* cluster of differentiation, *r-GPA* GPA patient in remission, *T*_*EM*_ effector memory T cell

Interestingly, the accumulating number of organs involved over the total disease course correlated negatively with the frequency of T_EM_1 cells (Spearman’s rho = -0.264, *p* = 0.037) but correlated positively with T_EM_17 cells (Spearman’s rho = 0.390, *p* = 0.002). In addition, generalized r-GPA patients showed lower frequencies of T_EM_1 cells in comparison to localized r-GPA patients (10.7%, 6.3–16.5% vs 17.9%, 12.6–20.4%, *p* = 0.016). The frequencies of T_EM_17 cells were higher in generalized r-GPA compared to localized r-GPA patients (24.8%, 19.2–30.8% vs 19.3%, 15.0–21.4%, *p* = 0.037). This indicates that r-GPA patients with more systemic manifestations have an increased T_EM_17-mediated immune response, whereas r-GPA patients with more local manifestations have a more T_EM_1-directed immune response. However, disease duration and total number of relapses did not correlate with either the frequencies of T_EM_1 cells or T_EM_17 cells. Of note, we observed that r-GPA patients that did encounter one or more relapses after diagnosis (1 ≥ relapse r-GPA, *n* = 43) had higher frequencies of circulating T_EM_17 cells and lower frequencies of circulating T_EM_1 cells in comparison to r-GPA that experienced no relapse (non-relapse r-GPA, *n* = 20) since diagnosis (Fig. 4).

### Interaction of CMV serostatus and age on the different T cell subsets

The results described above regarding the differences between r-GPA patients and HCs in percentage of CD4^+^T_EM_ cells, T_EM_1 and T_EM_17 cells may indicate a possible interaction between CMV and age, as both factors influence the T cell memory compartment. To analyze this interaction a linear regression was performed that included interaction of CMV and age. This analysis demonstrated that differences in CD4^+^T_EM_ cells, T_EM_1 cells, and T_EM_17 cells between r-GPA patients and HCs were not attributed to CMV serostatus and age (see Additional file 1). Moreover, differences in T_EM_1 between relapse r-GPA patients and non-relapse r-GPA patients were not affected by CMV serostatus and age. The difference in T_EM_17 cells between relapse r-GPA patients and non-relapse r-GPA patients was minimally influenced by CMV serostatus and age as the differences for both factors were borderline significant.

Thus, CMV and age did not influence the differences in expansion of CD4^+^T_EM_ cells, T_EM_1 cells, and T_EM_17 cells, in both r-GPA patients and HCs.

## Discussion

In this study, we aimed to determine the distribution of circulating CD4^+^ T_EM_ cell subsets based on chemokine receptor expression in GPA patients. We demonstrated a significant increase in the proportion of T_EM_17 cells with a concomitant decrease in the proportion of T_EM_1 cells in peripheral blood of patients with r-GPA. Increased proportions of T_EM_17 cells were more pronounced in r-GPA patients with systemic manifestations, whereas r-GPA patients with local manifestations showed a remarkable increase in T_EM_1 cells. Interestingly, CMV seropositivity appeared to modulate the disturbed balance of T_EM_1 and T_EM_17 cells in r-GPA patients.

The decreased proportions of T_EM_1 cells in r-GPA patients compared to HCs reflect an aberrant T_EM_1 response in patients. It has been demonstrated that GPA patients with active or localized disease show a polarization toward a T_H_1-type response [8, 11, 31]. These studies showed an abundant T_H_1 cytokine (IFN-γ) and chemokine (CCR5) pattern on circulating T cells, as well as in granulomatous lesions compared to patients in remission or with generalized disease [11, 31]. It has been suggested that the disturbed T_H_1 response might play a role in the initiation of GPA. The disease can progress into a generalized GPA with a less prominent T_H_1-type response. The majority of r-GPA patients included in this study present generalized disease with a median disease duration of 9.6 years. This might explain the decreased proportion of circulating T_EM_1 cells in our r-GPA patients. However, one may also argue that the relative decrease in circulating T_EM_1 cells is due to an increased tissue migration of these cells. In GPA patients with generalized disease it has been reported that renal lesions show polarization toward T_H_1 type-responses [32]. However, we did not observe an association of T_EM_1 cells with renal involvement in r-GPA patients.

Our results regarding the increase in T_EM_17 response in GPA patients are in line with previous reports on increased T_H_17-associated activity in these patients. It has been reported that antigen-specific T_H_17 cells are expanded in GPA patients, irrespective of disease activity and maintenance therapy [13, 33]. In addition, serum IL-17A levels are also found to be elevated in active GPA patients and remained elevated in GPA patients recovering from active disease [12]. In line with this result, we observed a sustained T_EM_17 expansion over a period of 6 months in our r-GPA patients. Altogether, the involvement of T_H_17 cells in the immunopathology in GPA appears to be well established, although presently it remains unclear which mechanisms initiate T_H_17 responses in GPA. Possible explanations for the expanded T_EM_17 population might be related to the presence of granulomas, or chronic nasal carriage of *S. aureus* in GPA.

Granulomas are sophisticated and highly organized structures that typically consist of a sphere of highly activated macrophages surround by T lymphocytes. They provide a specialized niche for macrophage-T cell interaction, contributing to the differentiation and maturation of T cells [34]. The pro-inflammatory cytokine environment in granuloma may contribute to the aberrant T_H_1 and T_H_17 cell distribution found in the circulation. Since, granulomas are common clinical manifestations in GPA patients they may provide an ideal environment for T_EM_17 cell expansion. Engagement of CD4^+^T_EM_ cells with IL-6/TGFβ-producing macrophages may promote CD4^+^T_EM_ cell differentiation into T_EM_17 cells. In addition, macrophages also secrete IL-23, which sustains the T_H_17 population. Indeed, elevated serum levels of TGFβ, IL-6, and IL-23 have been reported in GPA, and, importantly, elevated levels of IL-23 correlated with disease severity in patients with GPA [12].

Chronic carriage of *S. aureus* constitutes a risk factor for the development of exacerbations in GPA. We have previously shown that the frequency of chronic nasal carriage of *S. aureus* is higher in GPA patients compared to HC [30]. Moreover, it was shown that nasal *S. aureus* carriage is associated with increased risk of relapse [30, 35]. Staphylococcal superantigens act as potent immune stimulators for T cells, resulting in polyclonal T cell proliferation and pro-inflammatory cytokine production [36]. In vitro studies demonstrated that stimulating T cells with staphyloccal exotoxins (alpha-toxin and SEB), strongly induced IL-17A-secreting T cells [13, 37]. Therefore, the involvement of T_H_17 cells in GPA may possibly be driven by chronic nasal carriage of *S. aureus.* However, we did not observe increased frequencies of T_EM_17 cells in GPA patients carrying *S. aureus*. This observation is in line with earlier studies in GPA patients in which no correlation between the presence of staphylococcal superantigens and the expansion of T cell subsets in peripheral blood was found [27].

Remarkably, we observed that the proportion of T_EM_17 cells in r-GPA patients was highly associated with CMV serostatus with frequencies of T_EM_17 cells being decreased in CMV-seropositive r-GPA patients as compared to seronegative r-GPA patients. These observations indicate that latent infection with human CMV modulates the distribution of T_EM_ cell subsets, although the underlying mechanisms are unclear. For instance, CMV seropositivity is strongly associated with the presence of memory T cells. It has been demonstrated that only CMV-seropositive individuals possess significant numbers of CD4^+^CD28^-^ T cells and many of these T cells respond to CMV [38]. In fact, the expansion of CD4^+^CD28^-^ T cells in GPA is suggested to be driven by CMV infections, and is associated with increased risk of infection and mortality [15]. However, the precise role of CMV infection in T_H_1 and T_H_17 responses is poorly understood. Previous studies indicate that T cells expressing CXCR3 (T_H_1 type) arise during primary CMV infection and are maintained during latency [39]. In line with this study, we observed increased proportions of T_EM_1 cells in the circulation of CMV-seropositive r-GPA patients. The skewing toward a T_EM_1 response in CMV-seropositive r-GPA patients could also explain the decrease in the proportion of T_EM_17 cells since these two T_EM_ cells subsets inversely correlate with each other. Importantly, the difference in T_EM_1 cells between r-GPA patients and HCs was not influenced by CMV and age. Additionally, CMV serostatus did not influence the proportions of T_EM_1 cells in HCs whereas in r-GPA patients CMV serostatus had a major impact on both the proportions of T_EM_1, and T_EM_17 cells.

T_H_17 cells may also induce autoimmune responses. Very recently, it was shown that the frequency of T_H_17 cells (CCR6^+^) in rheumatoid arthritis (RA) patients is associated with anti-citrullinated protein antibodies (ACPA) status [28]. In particular, CCR6^+^ T_H_ cell proportions were higher in ACPA-positive RA patients in comparison to ACPA-negative RA patients, and inversely correlated with disease duration in ACPA-negative patients. If this were the case in GPA patients, one may argue that the increase in T_EM_17 cells might be associated with ANCA status and could be a tool to discriminate ANCA-positive patients from those that are ANCA-negative. In contrast to the data in RA patients, we did not observe any association regarding ANCA status with the frequency of T_EM_17 cells in r-GPA patients. This is possibly due to the fact that ANCA titers in GPA patients fluctuate during the disease course, whereas ACPA-positive RA patients consistently remain ACPA-positive over time. On the other hand, we found that T_EM_17 cells in GPA patients showed a positive association with organ involvement, whereas T_EM_1 cells were negatively associated with organ involvement. This suggests a more severe disease course in individuals with a high frequency of T_EM_17 cells. Furthermore, we observed that persistent T_EM_17 expansion is associated with a higher tendency to relapse.

The current study was designed as a cross-sectional study using peripheral blood of quiescent GPA patients and HCs. The main limitations are the lack of absolute lymphocyte counts and study samples from GPA patients with active disease. Therefore, the current data only provides observational information of proportions of circulating CD4^+^ T_EM_ cell subsets in r-GPA patients. Further studies are warranted to assess blood samples from patients during active disease and to study the distribution of infiltrated T_EM_ cell subsets in nasal and renal biopsies to elucidate distinct migratory capacities of T_EM_1 and T_EM_17 cells and to confirm their role in inflamed target tissues in GPA. Since T_EM_ cells also appear in the urine during active renal GPA disease, analysis of urine samples might aid in demonstrating which distinct T_EM_ subsets are possibly involved in renal injury.

## Conclusions

This study describes the distribution of circulating CD4^+^ T_EM_ cell subsets identified based on chemokine receptor expression in r-GPA patients without any in vitro manipulation. It demonstrates an aberrant balance between T_EM_1 and T_EM_17 cells in r-GPA patients, which is shown to be associated with severity of the disease in terms of organ involvement, and tendency to relapse. Interestingly, the imbalance between T_EM_1 and T_EM_17 cells is modulated in CMV-seropositive r-GPA patients. Accordingly, future T cell phenotype studies should take into account chronic viral infections (i.e. CMV) for CD4^+^T_EM_ subset characterization.

## Additional file

Additional file 1: Table S1. Linear regression analysis for percentages of CD4 + TEM cells, TEM1, and TEM17 cells between r-GPA patients and HCs. (PDF 208 kb)

## Abbreviations

ACPA: Anti-citrullinated protein antibodies
BVAS: Birmingham Vasculitis Activity Score
CCR: C-C chemokine receptor
CD: Cluster of differentiation
CMV: Cytomegalovirus
CRP: C-Reactive protein
CXCR3: CXC chemokine receptor 3
eGFR: Estimated glomerular filtration rate
ELISA: Enzyme-linked immunosorbent assay
ENT: Ear, nose and throat
GPA: Granulomatosis with polyangiitis
HC: Healthy control
IL: Interleukin
PR3-ANCA: Antineutrophil cytoplasmic antibodies targeting proteinase 3
RA: Rheumatoid arthritis
r-GPA: GPA patient in remission
*S. aureus*: *Staphylococcus aureus*
T_EM_: Effector memory T cell
T_H_: T helper
T_REG_: regulatory T cell
