# Supplementary material for: Chemokine receptor co-expression reveals aberrantly distributed TH effector memory cells in GPA patients
Source: Arthritis Res Ther. 2017 Jun 14;19:136. doi: 10.1186/s13075-017-1343-8 (PMC5471953; doi:10.1186/s13075-017-1343-8)
Supplement: Additional file 1: Table S1. — Linear regression analysis for percentages of CD4 + TEM cells, TEM1, and TEM17 cells between r-GPA patients and HCs. (PDF 208 kb) [file 13075_2017_1343_MOESM1_ESM.pdf]

Additional file 1

Table S1: Linear regression analysis for percentages of $CD4^{+}T_{EM}$ cells, $T_{EM}1$, and $T_{EM}17$ cells between r-GPA patients and HCs

	Predicting variable	B	(95% CI)	p-value
**$CD4^{+}T_{EM}$ cells**	Group (r-GPA and HCs)	17.3	(12,104 – 22,516)	<0.0001
	CMV and age	17.1	(12,161 – 21,969)	<0.0001
**Log(T_{EM}1) cells**	Group (r-GPA and HCs)	-.121	(-.219 – -.023)	0.016
	CMV and age	-.107	(-.198 – -.017)	0.021
**T_{EM}17 cells**	Group (r-GPA and HCs)	5,73	(2,321 – 9,132)	0.001
	CMV and age	5,29	(2,235 – 8,339)	0.001
**Log(T_{EM}1) cells**	Group (relapse and non-relapse r-GPA patients)	-.145	(-.288 – -.002)	0.047
	CMV and age	-.135	(-.263 – -.007)	0.040
**T_{EM}17 cells**	Group (relapse and non-relapse r-GPA patients)	4.80	(-.227 – 9.823)	0.061
	CMV and age	4.55	(.145 – 8.96)	0.043
